# Deficiency of the Cyclin-Dependent Kinase Inhibitor, CDKN1B, Results in Overgrowth and Neurodevelopmental Delay

**DOI:** 10.1002/humu.22314

**Published:** 2013-03-15

**Authors:** William Grey, Louise Izatt, Wafa Sahraoui, Yiu-Ming Ng, Caroline Ogilvie, Anthony Hulse, Eric Tse, Roman Holic, Veronica Yu

**Affiliations:** 1 Department of Medical & Molecular Genetics, King's College London School of Medicine, Guy's Hospital, Great Maze PondLondon, United Kingdom; 2 Clinical Genetics Department, Guy's and St Thomas' NHS Foundation TrustLondon, United Kingdom; 3 Department of Medicine, The University of Hong Kong, Queen Mary HospitalHong Kong; 4 Cytogenetics Department, Guy's and St Thomas' NHS Foundation TrustLondon, United Kingdom; 5 Department of Paediatrics, Evelina Children's Hospital, Guy's & St Thomas' NHS Foundation TrustLondon, United Kingdom; 6 Institute of Animal Biochemistry and Genetics, Slovak Academy of SciencesIvanka pri Dunaji, Slovakia

**Keywords:** CDKN1B, MEN syndrome, developmental delay, cancer

## Abstract

Germline mutations in the cyclin-dependent kinase inhibitor, *CDKN*1*B*, have been described in patients with multiple endocrine neoplasia (*MEN*), a cancer predisposition syndrome with adult onset neoplasia and no additional phenotypes. Here, we describe the first human case of *CDKN*1*B* deficiency, which recapitulates features of the murine *CDKN*1*B* knockout mouse model, including gigantism and neurodevelopmental defects. Decreased m*RNA* and protein expression of *CDKN*1*B* were confirmed in the proband's peripheral blood, which is not seen in *MEN* syndrome patients. We ascribed the decreased protein level to a maternally derived deletion on chromosome 12p13 encompassing the *CDKN*1*B* locus (which reduced m*RNA* expression) and a de novo allelic variant (c.-73*G*>*A*) in the *CDKN*1*B* promoter (which reduced protein translation). We propose a recessive model where decreased dosage of CDKN1B during development in humans results in a neuronal phenotype akin to that described in mice, placing *CDKN*1*B* as a candidate gene involved in developmental delay.

The cyclin-dependent kinase (CDK) inhibitor *CDKN1B* (or p27^Kip1^) (NM_004064.3, MIM #600778; see http://www.lovd.nl/CDKN1B) belongs to the Kip/Cip (kinase inhibitor protein/cyclin inhibitor protein) family of CDK inhibitors. Its primary role is to regulate cell cycle progression during the G1/S transition [Sheaff et al., 1997] by inhibiting cyclin A/CDK2 activity until the onset of S phase. *CDKN1B* has been identified as an important tumor suppressor gene. Low *CDKN1B* expression in tumors is associated with rapid cell cycle entry, a high proliferative index, and poor prognosis [Marinoni and Pellegata, 2012]. Germline mutations in *CDKN1B* have been reported in families with the multiple endocrine neoplasia (MEN) syndrome. Thus far, families with reported *CDKN1B* mutations present with a variant of the autosomal dominant MEN1 syndrome. This is characterized by at least two endocrine tumors typically involving the parathyroid and pituitary glands [Marinoni and Pellegata, 2012].

Because of the importance of *CDKN1B* in cancer, multiple mouse models have been created. The *CDKN1B* homozygous knockout mouse (hereby denoted *p27^−/−^*) is large and develops adenomas in the intermediate pituitary lobe [Fero et al., 1996]. Mice heterozygous for p27 have an increased susceptibility to both mammary and prostate tumors [Gao et al., 2004; Muraoka et al., 2006a]. The *p27^−/−^* mouse also exhibits gigantism and hyperplasia of multiple organs including the brain (this is not seen in heterozygote animals) [Fero et al., 1996]. This was initially believed to be due to impaired cell cycle exit of neuroprogenitors during development. However, further investigation revealed that p27 plays a more complex role in neuronal development [Nguyen et al., 2004], as *p27^−/−^* mice also display abnormal cortical neuron migration. Interestingly, the role of p27 in neuronal migration is independent of its interaction with CDKs and involves the control of neuron cytoskeleton reorganization through multiple signaling pathways [Nguyen et al., 2006b].

In this study, we report the first human subject to present with overgrowth, autism/severe developmental delay and reduced p27 expression. The proband (annotated II:1) was the child born at full-term by cesarean section to nonconsanguineous Bengali parents (pedigree depicted in Fig. 1A). Birth weight was 3.765 kg at 41 + 5 weeks (50th centile) and occipital frontal circumference of the head (OFC) was 36 cm (50–75th). He presented with significant developmental delay, overgrowth, and autism (both OFC and height above 98th centile and out of keeping with family size from the first year of life; Supp. Fig. S1). At the age of 33 months, his language and manipulation skills were at the 15 month level but his locomotor skills were age appropriate. His weight was 23.1 kg (above the 99.6th centile), height was 104.2 cm (99.6th centile, with mid-parental centile for height at 25th centile), and head circumference was 55.2 cm (99.6th centile, parental OFCs were at 25–50th and 2–9th centiles, respectively).

**Figure 1:**
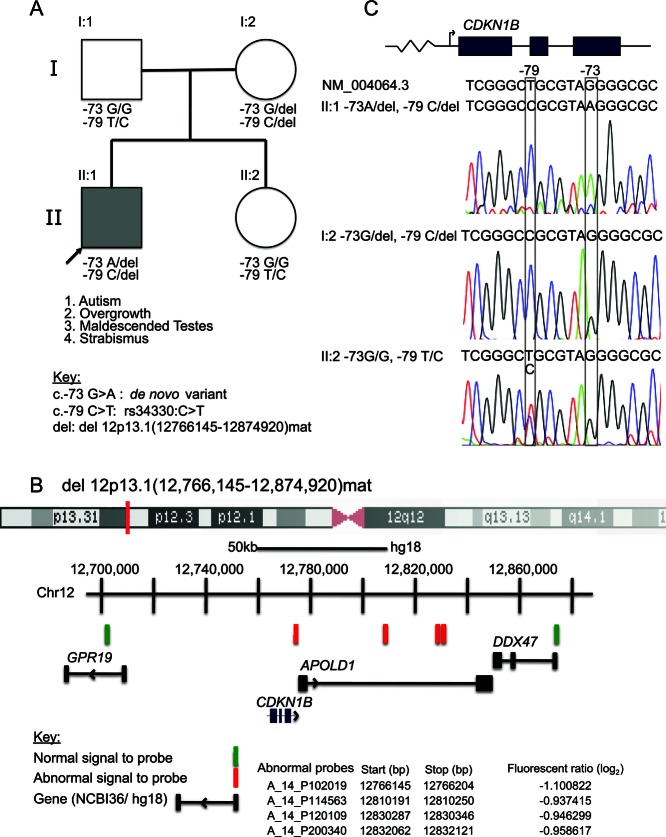
**A**: Pedigree of the nonconsanguineous Bengali family. The proband (II:1) presented with overgrowth, autism, and severe developmental delay with absent speech at the age of 3 years. Array CGH demonstrated a deletion on 12p13.1 in the proband and his mother (I:2). Sanger sequencing of the *CDKN*1*B* gene demonstrated a SNP, rs34330: −79C>T, in the proband's father (I:1) and his sister (II:2). The proband harbors a de novo variant that is not seen in other members of the family at c.−73G>A. **B**: Array CGH analysis. Diagrammatic depiction of the maternally derived 12p13.1 deletion identified by array CGH (Agilent AMAID 017457). Coordinates displayed in build NCBI36/hg18. The position of probes showing normal copy number is shown in green, probes showing abnormal copy number are shown in red. Probe names, base-pair coordinates, and fluorescent ratios for the abnormal probes are shown (bottom right). For a patient: reference ratio of 1:2 (one allele deleted in the patient), the theoretical log_2_ value is −1. **C**: rs34330:C>T and c.−73G>A. Bidirectional Sanger sequencing of the promoter region of *CDKN*1*B* revealed the rs34330:C>T (−79C>T) in the proband's father and sister (I:1, II:2) (representative sequencing trace shown). In the reference sequence, the ancestral allele of T is shown but variant at this site is common in human populations (Supp. Fig. S3). The T allele has been linked to cancer [Chang, 2004; Landa et al., 2010]. A previously unreported SNP c.−73G>A was seen in the proband only (II:1). Sequencing primers are detailed in Supp. Figure S4A.

At last review, aged 5 years 6/12, he still had no speech, was not toilet-trained, and had challenging behavior due to severe autism. Other features included a left-sided strabismus and maldescended testes. His height was 119.5 cm (91–98th centile for height), 56.5 cm (>98th centile for OFC) and 29.8 kg (>99.6th centile for weight). In comparison, his younger sister was on 25th centile for all growth parameters and has grown proportionally along these centiles from birth, in keeping with familial stature.

When assessed for possible causes of developmental delay, a full panel of tests (including thyroid function tests, CK, plasma amino acids, urinary organic acids, and white cell enzymes) showed no abnormalities. No atypical transferrin glycoforms were found and there was no evidence of a purine or pyrimidine disorder. Fragile X test was also normal.

Blood was therefore taken at the genetics clinic from the proband, his unaffected sister and parents for DNA extraction. Array CGH using a custom Agilent oligonucleotide array (comprising 44,000 probes across the genome) detected a maternally inherited interstitial deletion of the short arm of chromosome 12. Figure 1B shows the putative deletion interval chr12:12,766,145-12,874,920 (hg18), which includes the 5′ end of *CDKN1B*, *APOLD1*, and the 5′ untranslated end of *DDX47*. Because of limited resolution of the array CGH, we carried out Sanger sequencing and this confirmed that *CDKN1B* was deleted in its entirety on the maternal allele, with the 5′ breakpoint mapped outside the coding region of *GPR19* (data not shown).

The *APOLD1* gene (MIM #612456) encodes a vascular early response gene [Regard et al., 1997] that has not previously been reported to be mutated in human disease. Likewise, *DDX47* is a probable ATP-dependent RNA helicase not linked to human disease. Although not possible to exclude potential roles of these genes secondary to haploinsufficiency (Supp. Fig. S2) in accounting for the patient's phenotype, based on current biological knowledge available, *CDKN1B* is the most likely candidate, as there already exists a plethora of data in animal models to support its role in neurogenesis.

We hypothesized that deletion of *CDKN1B* may be causal to the neurological phenotype seen in the proband. We noted that although the 12p13.1 deletion was maternally inherited, the proband's mother (annotated I:2) was intellectually normal. Given the known role of CDKN1B in neurodevelopment, we proceeded to examine the proband's paternal allele.

Sanger sequencing revealed a previously reported SNP, rs34330:C>T (-79C>T) in the proband's intellectually normal father (I:1) and sister (II:2) (Fig. 1C) in the promoter region of *CDKN1B*. The rs34330:C>T SNP has been shown to have a significant association with prostate cancer predisposition [Chang, 2004] and an increased risk of thyroid cancer in the Spanish population [Landa et al., 2010]. This SNP was not observed in the proband or his mother (I:2). Further sequencing revealed that the proband harbored a de novo variant c.-73G>A, close to rs34330 (Fig. 1C and Supp. Fig. S3). No other mutations were detected in the remainder of the *CDKN1B* coding sequence in the proband (II:1).

To determine if the maternally derived 12p13.1 deletion in combination with the de novo polymorphism at the promoter altered *CDKN1B* expression in our proband, we analyzed *CDKN1B* mRNA and protein levels in peripheral blood (Fig. 2A). RNA was extracted from peripheral blood, reverse transcribed, and analyzed by quantitative PCR (Fig. 2A). Compared with the proband's unaffected sister (II:2), both the proband (II:1) and his unaffected mother (I:2) harbor a four-fold decrease in *CDKN1B* mRNA expression. We proceeded to test if the reduction in mRNA expression translated into a significant decrease in protein expression. Western blotting on proteins extracted from peripheral blood showed that the proband (II:1) had significantly decreased protein expression of CDKN1B compared with his sister (II:2) and mother (I:2) (Fig. 2B). This could not be explained by reduced mRNA level alone, as the proband has decreased mRNA expression similar to his mother, but the low mRNA in the mother did not result in significantly decreased protein expression. It appears therefore that heterozygous deletion of *CDKN1B* on the maternal allele significantly altered RNA expression but it was the combination of this and the -73G>A SNP at the promoter that resulted in significantly decreased CDKN1B protein level in the proband. To investigate the potential role of the -73G>A SNP in contributing to reduced protein translation, we employed a luciferase reporter assay (Fig. 2C). Landa et al. (2010) previously reported that the -79T allele results in reduced protein translation in a luciferase reporter assay. Consistent with this, we observed that the -73A allele also confers a similar reduction in luciferase activity (Fig. 2C).

**Figure 2:**
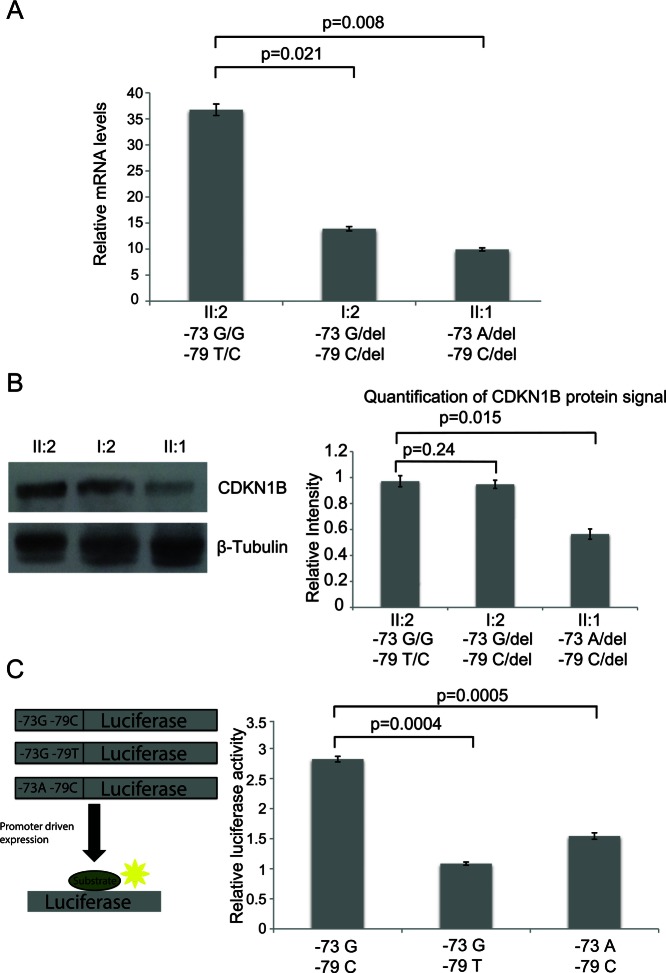
**A**: Quantification of CDKN1B mRNA expression by qPCR. Total RNA was extracted from peripheral blood. White blood cells from peripheral blood were spun down, lysed, and total RNA was purified using the Qiagen RNA easy extraction kit (cat. 52304). RNA was reversed transcribed, and quantitative PCR was carried using the Qiagen one-step SYBR green RT-PCR kit. Q-PCR primers (primer sequences are detailed in Supp. Fig. S4B) spanned the 3′ end of exon 1 (forward) of *CDKN*1*B* to the 5′ end of exon 2 (reverse). The amount of *CDKN*1*B* detected was normalized against the ubiquitously expressed *ABL1* gene and expressed as a relative value Ratio (*ABL1*/*CDKN1B*) = 2^CT(*ABL1*)-CT(*CDKN1B*)^. **B**: Quantification of CDKN1B minimal promoter activity. Region incorporating the 5′UTR of *CDKN*1*B* (encompassing the −79 and −73 residues) were amplified from patient DNA and subcloned into the pGL3 basic luciferase reporter vector (Promega cat. E1751). One microgram of pGL3^−79C-73G^, pGL3^−79T-79G^, pGL3^−79C-73A^, or an empty pGL3 vector was cotransfected with 20 ng of pRL-TK (Renilla reporter construct, Promega cat. E2241) into HEK293T cells with the ProFection mammalian transfection system (Promega cat. E1200) according to the manufacturer's protocol. Cells were transfected for 48 hr, followed by a 12 hr period of serum starvation. The dual-luciferase reporter assay system (Promega cat. E1910) was used to assay relative luciferase activity. The data represent the mean of three independent experiments with standard error bars. **C**: Western blot analysis of CDKN1B from white blood cells. Protein samples were extracted from peripheral blood samples by first isolating white blood cells through centrifugation. These cells were subsequently lysed using RIPA lysis buffer (50 mM Tris HCl pH 7.4, 1% NP40, 0.25% sodium deoxycholate, 150 mM NaCl, 1 mM EDTA) and run on a 12% SDS-PAGE. Western blotting was carried out using an anti-p27 antibody (BD Biosciences cat. 610242) and an anti-β-tubulin antibody (Cell Signaling cat. 2128) (left). Relative intensity of the signal obtained from the western blot was quantified using ImageJ (NIH). Bar chart shows the average of signals (and standard error) quantified from three blots normalized to β-tubulin level in each independent blood sample (right).

Millard et al. (2000) previously reported a U-rich element in the 5′ untranslated region in the *CDKN1B* promoter that is necessary for translation of p27 mRNA independent of mRNA stability. The -73G>A SNP is located in an evolutionarily conserved region just upstream of the U-rich region (Supp. Fig. S3) and is shown to be involved in binding to HuR, an RNA binding protein that modulate mRNA translation. Mechanistically, we postulate a recessive model similar to one proposed by Landa et al. that the -73G>A variant contributes to decreased translation of CDKN1B at protein level only in a homozygous setting (in our proband, due to deletion of the maternal allele). Consistent with this, we found that the heterozygote -79C/T allele in the sister, II:2, conferred a statistically insignificant reduction in mRNA level when compared with a control sample with a -79C/C genotype (data not shown) and resulted in no change to protein level.

Taken together, we postulate that the neurological phenotype in the proband fits a recessive mode of inheritance. It is impossible to determine retrospectively when the de novo -73A arose in our proband during embryonic development. The mutation could be postzygotic or somatic. Nevertheless, we believe that both the reduced mRNA transcription secondary to the 12p13.1 deletion and the -73A genotype on the remaining allele were necessary to reduce overall p27 protein to below a biological threshold to result in the observed clinical phenotypes. CDKN1B expression is meticulously controlled during development [Goto et al., 2004; Kiyokawa et al., 1996]. We hypothesize that a given threshold of CDKN1B expression is necessary to ensure normal neurodevelopment. In the initial report in mouse models, gigantism and multiorgan hyperplasia in the p27 homozygote knockout are not caused by changes in pituitary growth hormone levels at early stages [Fero et al., 1996]. Recent work has pinpointed the effect of p27 absence and gigantism to increased Sox2 levels, as p27 directly inhibits Sox2 expression [Li et al., 2012]. In *p27^−/−^* mice, the gigantism was due to Sox2-dependent increase in thickness of the pituitary progenitor layer. This layer constitutes the origin of pituitary adenomas over time. There was currently no clinically detectable evidence of organomegaly in our proband but endocrine studies revealed a consistently elevated level of (insulin-like growth factor 1) IGF-1 (over 95th centile for age). This could be secondary to the presence of an early pituitary microadenoma, yet undetectable by MRI, and might contribute to the overgrowth phenotype.

Among the family of the Kip/Cip CDK inhibitors, *CDKN1C* or p57^KIP2^ (MIM #600856) is closely related to *CDKN1B*. p57 knockout mice share similar phenotype to the p27 knockout mouse including macrocephaly and cortical hyperplasia in the brain [Mairet-Coello et al., 2012]. Germline heterozygote mutations in human *CDKN1C* are related to Beckwith–Wiedemann Syndrome, which is associated with overgrowth, developmental delay and tumor predisposition [Zhang et al., 1997]. Our study suggests the closely related CDK inhibitor, *CDKN1B*, may result in a similar phenotype if protein expression is below a certain threshold. Further studies are warranted to delineate how commonly *CDKN1B* mutations are involved in developmental delay and whether all these cases are similar to the result of reduced CDKN1B protein expression.

## Supplementary material

Disclaimer: Supplementary materials have been peer-reviewed but not copyedited.
