# Supplementary material for: Deficiency of the Cyclin-Dependent Kinase Inhibitor, CDKN1B, Results in Overgrowth and Neurodevelopmental Delay
Source: Hum Mutat. 2013 Mar 15;34(6):864–8. doi: 10.1002/humu.22314 (PMC3708111; doi:10.1002/humu.22314)
Supplement: Supplementary file 1 [file humu0034-0864-SD1.pdf]

Supporting Information for the article:

Deficiency of the cyclin-dependent kinase inhibitor, CDKN1B, results in overgrowth and neurodevelopmental delay

William Grey, Louise Izatt, Wafa Sahraoui, Yiu-Ming Ng, Caroline Ogilvie, Anthony Hulse, Eric Tse, Roman Holic , and Veronica Yu

Index of Supp. Figures S1-S4

Supp. Figure S1. Growth chart of the proband

Supp. Figure S2. mRNA expression of *APOLD1* and *DDX47*

Supp. Figure S3. Details of the *CDKN1B* 5'UTR region

Supp. Figure S4. Primer sequences

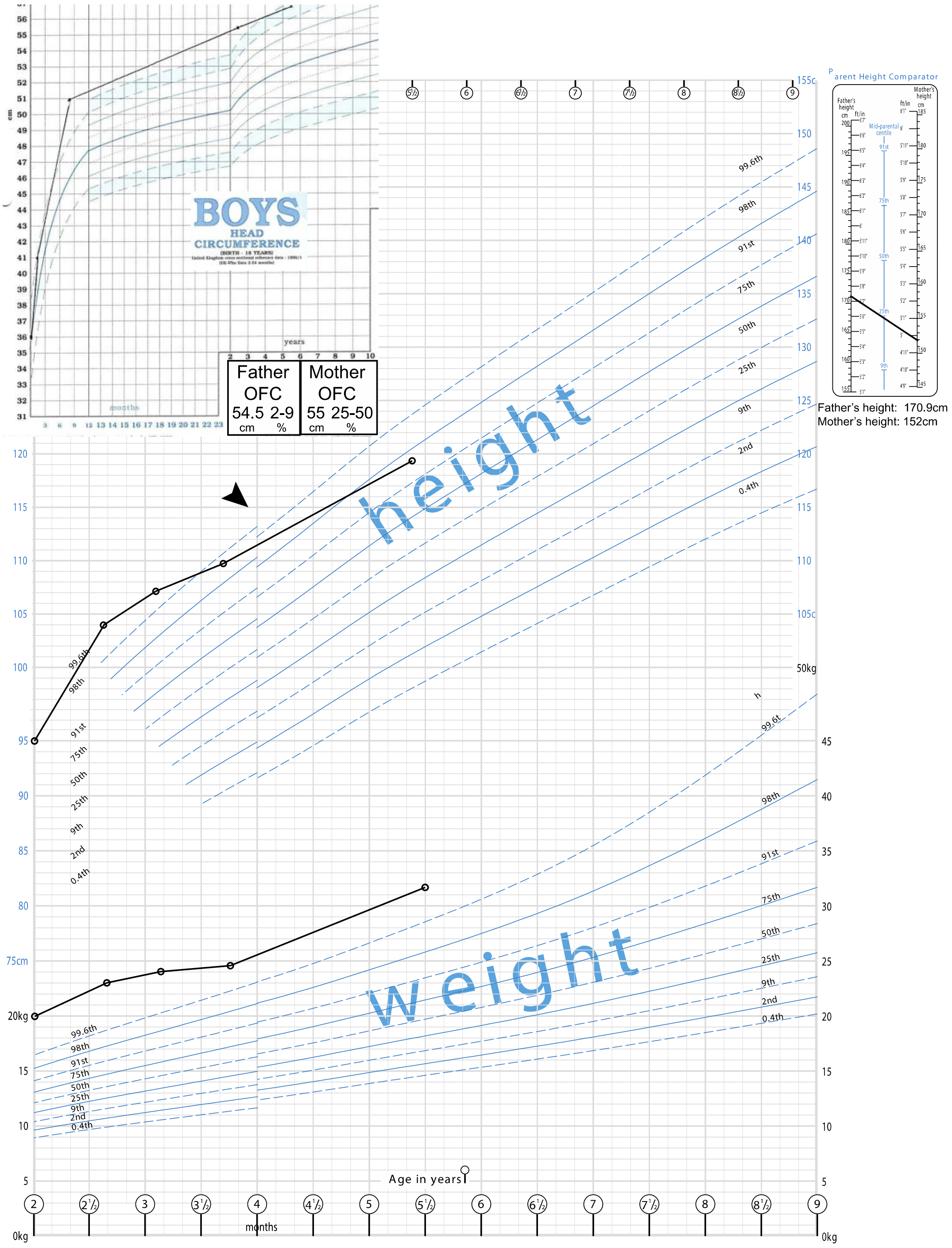

Supp. Figure S1. Growth chart of the proband. (Arrowhead indicates Transit point from UK-WHO to UK90 data). The proband's (II:1, -79C/del -73A/del) birth weight was 3.765 kg (50th centile), with a head circumference (OFC, top left) of 36cm (50-75th centile).

Symmetrical overgrowth was observed in all growth parameters from the first year of life. At age 33 months, the proband's head circumference was larger than his parents (OFC of above 99.6th centile).

Top right: Mid-parental height (MPH) predicts 25th centile for the proband’s growth parameters (which is the growth his younger, unaffected sister (II:2, -79T/C -73G/G)). Currently at aged 5 yr and 6 months, the proband’s growth parameters are as follows: 56.5cm OFC (>98th), height 119.5cm (91-98th), weight 29.8kg (>99.6th).

Further assessment of the proband aged 3yrs 9/12 and 5yr 6/12 saw continued large stature and poor social communication skills due to autism. He has no recognizable single words aged 5 years and has severe learning difficulties and challenging behaviour. He is placed in a school for children with autism and has a statement to support his special educational needs.

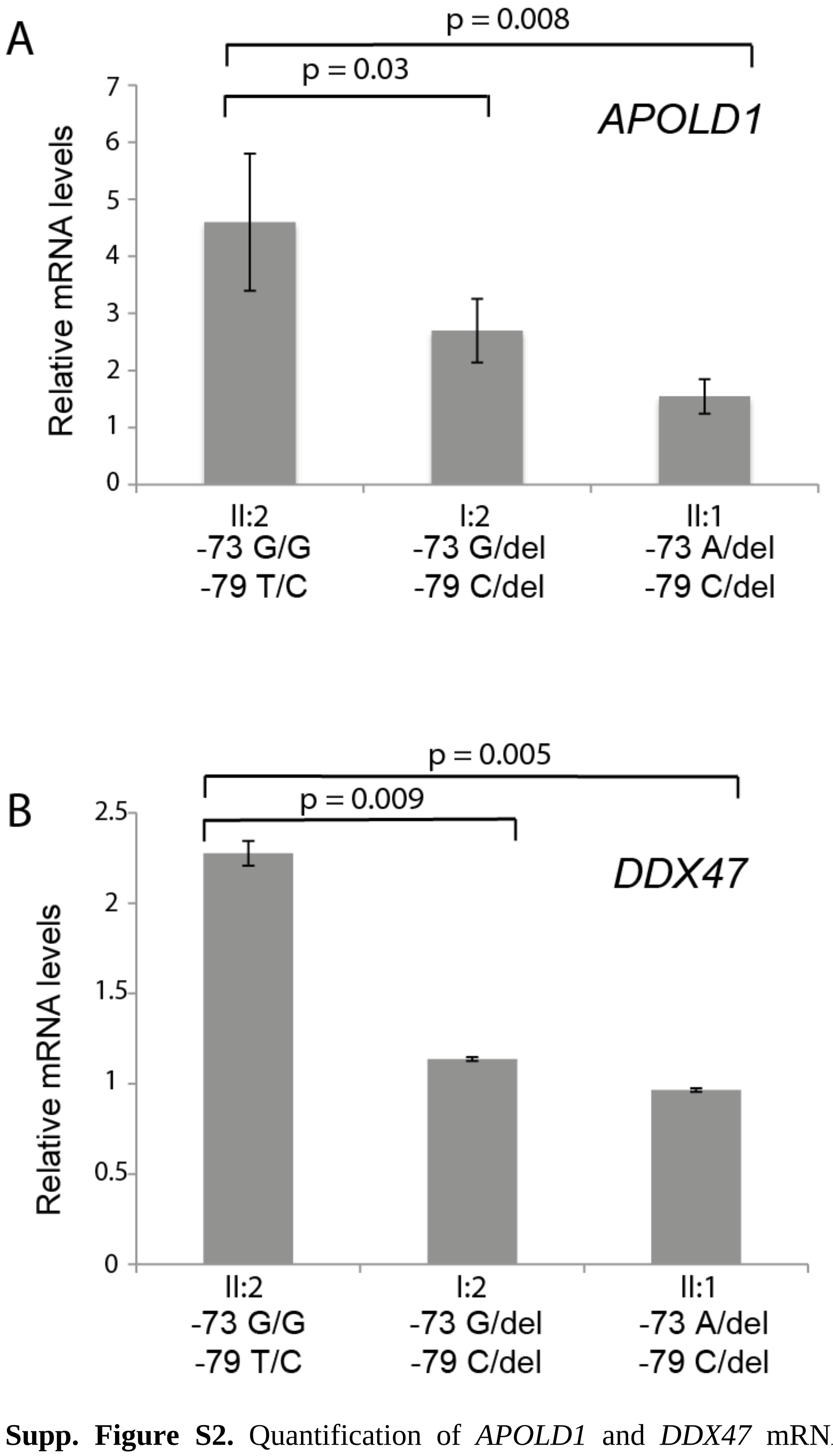

Supp. Figure S2. Quantification of *APOLD1* and *DDX47* mRNA expression by qPCR. RNA extraction and quantitative PCR were carried out as described in Figure 2A. Primers used were as follows; *APOLD1* forward (CCACGACCTCAAGATCTCTG), *APOLD1* reverse (AGCTTGGTGTTTTCCTAAGGG); *DDX47* forward (GAATATGGATTTTGAGACAGAGGTTG), *DDX47* reverse (CGGCACATTTCACAGGATTC). The amount of gene expression was normalised against *ABL1* expression and expressed as a relative value as described in Figure 2. Error bars represent triplicates of 3 independent experiments.

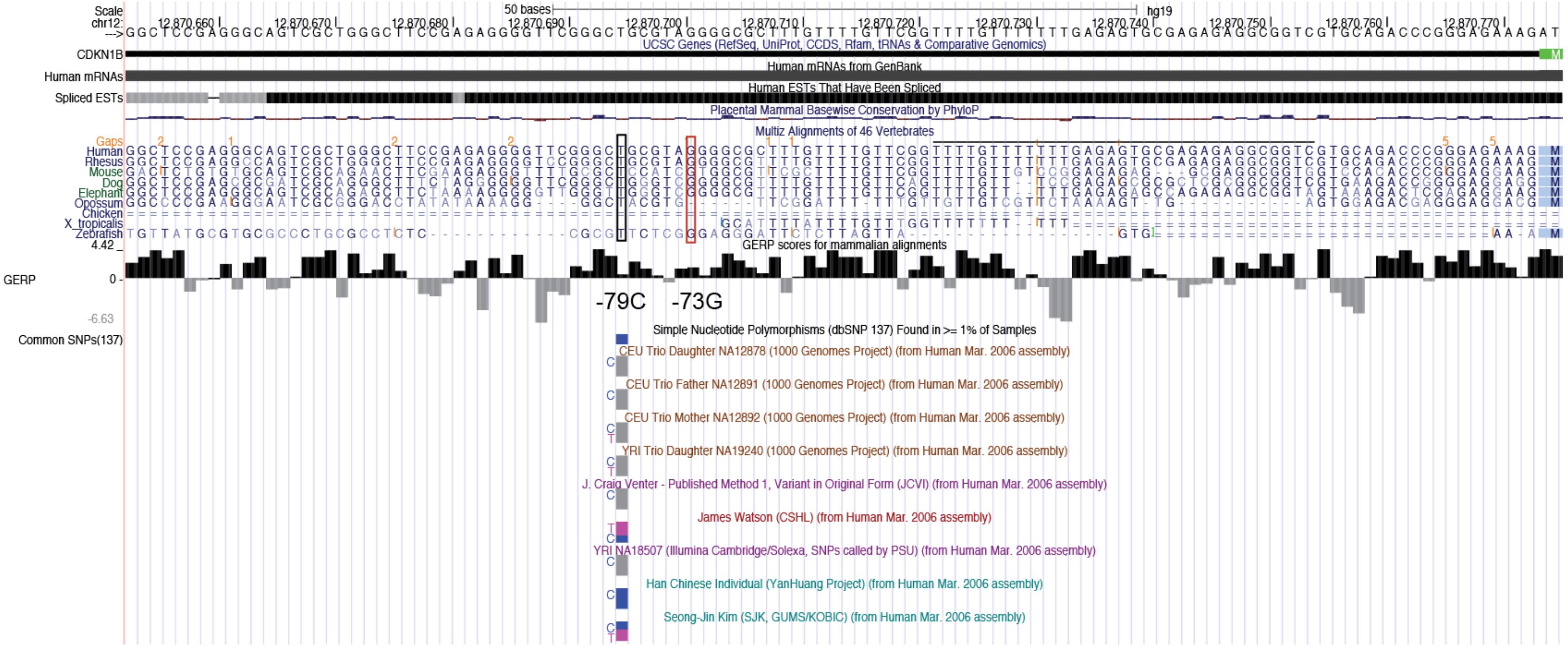

Supp. Figure S3. Details of the *CDKN1B* 5'UTR region. Details of the 5'UTR region of the *CDKN1B* gene up to position of the first methionine (far right, blue box) depicted by the UCSC genome browser (GRCh37/hg19). The U-rich region is underlined in black. Alignment of equivalent regions from other eukaryotic species are shown, with GERP score. The -79C and -73G positions are boxed in black and red respectively. Polymorphisms previously described at the -79T position in human populations are detailed beneath the alignment. For the -73G position, the GERP score has an average value of: 1.22727 min -2.31 max 3.56, standard deviation 1.69505. The -73A variant has been submitted at curated at http://www.lovd.nl/CDKN1B.

A

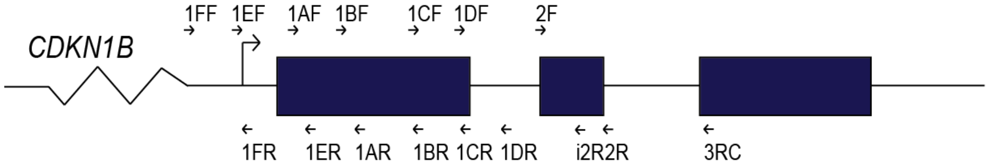

Name	Sequence (5'-3')	Strand	Forward/Revers	GC%	Tm(°C)	Source
Hp27_1AF	GTCGGGGTCTGTGTCTTTTG	PLUS	FORWARD	55	64.5	Costa-Guda et al. (2011)
Hp27_1AR	CCATGTCTCTGCAGTGCTTC	MINUS	REVERSE	55	63.8	Costa-Guda et al. (2011)
Hp27_1BF	TGTCTAACGGGAGCCCTAGC	PLUS	FORWARD	60	65.4	Costa-Guda et al. (2011)
Hp27_1BR	AGTAGAACTCGGGCAAGCTG	MINUS	REVERSE	55	63.4	Costa-Guda et al. (2011)
Hp27_1CF	AGTTAACCCGGGACTTGGAG	PLUS	FORWARD	55	64	Costa-Guda et al. (2011)
Hp27_1CR	GTCCGACGGATCAGTCTTTG	MINUS	REVERSE	55	64.7	Costa-Guda et al. (2011)
Hp27_1DF	AGGAGAGCCAGGATGTCAGC	PLUS	FORWARD	60	65.9	Costa-Guda et al. (2011)
Hp27_1DR	GCCAGGTAGCACTGAACACC	MINUS	REVERSE	60	64.7	Costa-Guda et al. (2011)
Hp27_1EF	ACTCGGACGGGCTTTGCCAC	PLUS	FORWARD	50	50	This study
Hp27_1ER	GAACAAAACAAAGCGCCCCT	MINUS	REVERSE	65	68	This study
Hp27_1FF	CTTCTTCGTCAGCCTCCCTT	PLUS	FORWARD	55	64	This study
Hp27_1FR	CAAGCGGAGAGGGTGGCAAA	MINUS	REVERSE	60	65	This study
Hp27_2F	CTGACTATGGGGCCAACTTC	PLUS	FORWARD	55	63.4	Costa-Guda et al. (2011)
Hp27_2R	GCCAGCAACCAGTAAGATCAG	MINUS	REVERSE	52.3	63.7	Costa-Guda et al. (2011)
Hp27_i2R	TTAATTTGCCAGCAACCAGTA	PLUS	FORWARD	38	62	Costa-Guda et al. (2011)
Hp27_2AF	AAATGCCGGTTCTGTGGAGC	PLUS	FORWARD	55	64	This study
Hp27_3RC	CATTCCATGAAGTCAGCGATA	MINUS	REVERSE	42.8	62.6	Costa-Guda et al. (2011)
Hp27_u1F	TTGAAGGTGGCTCTGTCATC	PLUS	FORWARD	50	52	This study
Hp27_u1R	TTGCCTTCTTTCCCAGTAGC	MINUS	REVERSE	50	52	This study
Hp27_u2F	GACAGGGGAATTTCATGTTG	PLUS	FORWARD	45	49	This study
Hp27_u2R	GTCCCACTGTGGTGTTCATC	MINUS	REVERSE	55	53	This study
Hp27_u3F	GGGTTCTCTGTCTCACACTT	PLUS	FORWARD	50	51	This study
Hp27_u3R	GAAGAAATATTTGTCAGAGTCC	MINUS	REVERSE	36	47	This study
Hp27_u4F	TTCCAACCTCTAGTTTTTGTG	PLUS	FORWARD	38	48	This study
Hp27_u4R	CTTTGAGAGAAGCCCTGAAG	MINUS	REVERSE	50	50	This study
Hp27_u5F	ACACTTCATTTGCTCTTTCCCC	PLUS	FORWARD	45	53	This study
Hp27_u5R	CAAATATTTACGGAGCACC	MINUS	REVERSE	42	46	This study
Hp27_u6F	CACTCAATAAATATTTGTTGAAAGA/	PLUS	FORWARD	25	50	This study
Hp27_u6R	GTGAAAAAAAAATACTATGGAG	MINUS	REVERSE	29	43	This study
Hp27_u7F	TTGCAGAAGGATGGCGCTCT	PLUS	FORWARD	55	56	This study
Hp27_u7R	GTTTTCAAGTCAGGACTTCC	MINUS	REVERSE	45	48	This study
Hp27_u8F	ATGAGACCTCTTGCTTGATC	PLUS	FORWARD	45	49	This study
Hp27_u8R	CGGTACCCATCATCTTGGTT	MINUS	REVERSE	50	51	This study
Hp27_u9F	ATATTAGAACGGAAGGTTGG	PLUS	FORWARD	40	47	This study
Hp27_u9R	ACTTATCATGAACTCAAGCTC	MINUS	REVERSE	38	48	This study
Hp27_u10F	GGGACTTGAGAGACTAGAGT	PLUS	FORWARD	50	49	This study
Hp27_u10R	GCTGACGAAGAAGAAAATGA	MINUS	REVERSE	40	48	This study
Hp27_u11F	GCCGCAACCAATGGATCTCC	PLUS	FORWARD	60	56	This study
Hp27_u11R	CCGTTAGACACTCGCACGTT	MINUS	REVERSE	55	55	This study

B

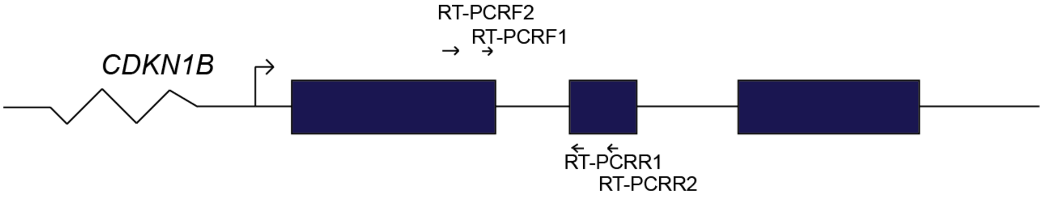

Name	Sequence (5'-3')	Strand	Forward/Revers	GC%	Tm(°C)	Source
Hp27_RT-PCR-F1	GGGTTAGCGGAGCAATGC	PLUS	FORWARD	61	63	This study
Hp27_RT-PCR-R1	TGTTCTGTTGGCTCTTTTGTTT	MINUS	REVERSE	36	62	This study
Hp27_RT-PCR-F2	TCTGAGGACACGCATTTGG	PLUS	FORWARD	52	62	This study
Hp27_RT-PCR-R2	TGTTCTGTTGGCTCTTTTGTTT	MINUS	REVERSE	36	62	This study

Supp. Figure S4. Primers used in this study for (A) sequencing *(Costa-Guda, et al., 2011)* and (B) RT-PCR of *CDKN1B.*

Supp. Reference

Costa-Guda J, Marinoni I, Molatore S, Pellegata NS, Arnold A. 2011. Somatic mutation and germline sequence abnormalities in CDKN1B, encoding p27Kip1, in sporadic parathyroid adenomas. J Clin Endocrinol Metab 96(4):E701-6.
